# Gut microbiome–immune–metabolic mechanisms in cerebrovascular disease: evidence-graded insights from cerebral small vessel disease, ischemic stroke, and intracerebral hemorrhage

**DOI:** 10.3389/fmicb.2026.1927904

**Published:** 2026-08-18

**Authors:** Chunna Ren, Yunxia Xiu, Yang Zhang, Xiaoyu Wang, Hezhen Zhao, Jiahui Tang, Qingjun Li, Shiwei Zhang, Feipeng Zhao

**Affiliations:** 1 Second Clinical Medical School, Mudanjiang Medical University, Mudanjiang, China; 2 Second Affiliated Hospital of Mudanjiang Medical University, Mudanjiang, China; 3 First Clinical Medical School, Mudanjiang Medical University, Mudanjiang, China; 4 School of Basic Medical Sciences, Mudanjiang Medical University, Mudanjiang, China; 5 Daqing People’s Hospital, Daqing, China

**Keywords:** cerebral small vessel disease, cerebrovascular disease, gut microbiome, host–microbe interaction, intestinal barrier, intracerebral hemorrhage, ischemic stroke, microbial metabolites

## Abstract

Cerebrovascular disease is increasingly being examined in relation to the gut microbiome, but the field has not advanced evenly across disease phenotypes. A central challenge is to distinguish broad dysbiosis-based associations from microbial functions, host-facing metabolites, epithelial barrier injury, and immune pathways that may plausibly influence neurovascular vulnerability or recovery. This distinction is particularly important because cerebral small vessel disease, acute ischemic stroke, and intracerebral hemorrhage differ in time scale, vascular pathology, clinical exposure, and available microbiome evidence. This review evaluates gut microbiome–immune–metabolic mechanisms across these cerebrovascular contexts with a focus on microbial ecology, intestinal barrier dysfunction, microbial translocation, short-chain fatty acids (SCFAs), trimethylamine N-oxide (TMAO), bile acid derivatives, tryptophan-linked metabolites, lipopolysaccharide (LPS)-related inflammatory signaling, and emerging multi-kingdom signals, including the gut virome and mycobiome. Current evidence is most convincing in acute ischemic stroke, where human cohort studies and experimental perturbation models link microbiome disruption, microbial metabolites, immune programming, and functional outcome. Evidence for imaging-defined cerebral small vessel disease remains more limited and is largely cross-sectional, whereas intracerebral hemorrhage is an emerging but mechanistically distinct domain. Virome- and mycobiome-related mechanisms remain exploratory and require longitudinal, multi-omics, and perturbation-based validation. This review argues that cerebrovascular microbiome research should move beyond taxonomic association toward time-resolved microbial function, host-facing metabolites, disease-specific host–microbe interfaces, and experimentally testable mechanisms. Candidate microbiome-directed interventions—including dietary, prebiotic, probiotic, postbiotic, fecal microbiota transplantation, defined microbial consortia, metabolite-targeted, and phage-based approaches—remain investigational. Their translation will require disease- and time-window-specific evaluation of biological target engagement, safety, and clinically meaningful outcomes. Longitudinal multi-omics cohorts, disease-specific models, and careful control of diet, antibiotics, vascular medications, hospitalization, and frailty will be essential for determining which gut microbiome-related pathways are causal, context-specific, modifiable, and therapeutically actionable.

## 1 Introduction

The vertebrate gut microbiome is a complex and dynamic microbial ecosystem that participates in host metabolism, mucosal immune education, epithelial barrier regulation, and systemic inflammatory homeostasis. Rather than acting only as a digestive compartment, the intestinal microbiome functions as a host–microbe interface through which microbial communities, structural components, and metabolites can influence distal organs. Experimental studies have shown that gut microorganisms regulate blood–brain barrier (BBB) integrity and microglial maturation, indicating that intestinal microbial communities can affect central nervous system immune and barrier biology (Braniste et al., 2014; Erny et al., 2015). These findings provide a mechanistic basis for considering cerebrovascular disease not only as a vascular or neurological disorder, but also as a condition potentially shaped by microbial ecology, metabolite-mediated signaling, and host–microbe immune interaction (Schächtle and Rosshart, 2021).

Aging is a particularly important context for this interaction. With increasing age, gut microbial communities may lose resilience, shift in taxonomic and functional composition, and become associated with impaired epithelial barrier regulation and chronic low-grade inflammation. Transfer experiments using aged microbiota have shown that microbial communities from older hosts can promote systemic inflammatory features in young germ-free recipients, supporting the concept that age-related dysbiosis may contribute actively to inflammaging rather than merely reflect chronological aging (Fransen et al., 2017). Because cerebral small vessel disease, ischemic stroke, and intracerebral hemorrhage occur predominantly in older adults, aging-related microbiome instability may form a biological background that modifies neurovascular vulnerability, immune tone, and recovery potential.

Cerebrovascular disease spans chronic and acute vascular phenotypes with different time scales and mechanisms. This disease grouping is also clinically relevant in Asian populations, where ischemic stroke and intracerebral hemorrhage both contribute substantially to stroke-related disability and mortality (Venketasubramanian, 2025; Tan et al., 2024; Wang et al., 2026). Cerebral small vessel disease (CSVD) develops gradually and is typically recognized through imaging-defined markers such as white matter hyperintensities, lacunes, cerebral microbleeds, and enlarged perivascular spaces (Wardlaw et al., 2013; Duering et al., 2023). Acute ischemic stroke and intracerebral hemorrhage (ICH), in contrast, present as abrupt vascular events but occur on the background of heterogeneous vascular aging, metabolic disease, immune status, medication exposure, diet, and prior microbial conditioning. These conditions should therefore not be treated as microbiome-equivalent disease states. Instead, they provide distinct models for examining how gut microbial ecosystems may contribute to chronic neurovascular vulnerability, acute barrier disruption, inflammatory amplification, and post-injury repair.

Among cerebrovascular conditions, acute ischemic stroke currently provides the most developed mechanistic evidence for microbiome involvement. Experimental work has shown that commensal microbiota can influence ischemic stroke outcome through intestinal immune programming, including modulation of regulatory T cells, IL-17-positive γδ T cells, and immune-cell trafficking from gut-associated compartments toward injured brain tissue (Benakis et al., 2016; Singh et al., 2016). Additional studies indicate that ischemic stroke can rapidly induce gut dysbiosis, including expansion of potentially pro-inflammatory taxa such as Enterobacteriaceae, and that this stroke-induced dysbiosis may further exacerbate brain infarction (Xu et al., 2021). Human studies have also proposed gut dysbiosis signatures associated with stroke severity, brain injury, and clinical prognosis (Xia et al., 2019). These observations support a reciprocal brain–gut–microbiome model in which acute cerebral injury changes intestinal physiology and microbial community structure, while altered microbial signals feed back to influence secondary neuroinflammation and injury progression.

Microbial metabolites and microbial structural products provide a second mechanistic layer linking gut ecology to cerebrovascular injury. Short-chain fatty acids (SCFAs), trimethylamine N-oxide (TMAO), and lipopolysaccharide (LPS)-related signals have been associated with intestinal barrier integrity, endothelial activation, immune-cell polarization, BBB function, and functional outcome after stroke. Reduced SCFA-producing bacteria and lower fecal SCFA levels have been associated with increased stroke severity and poor functional outcome after acute ischemic stroke (Tan et al., 2021). Gut microbial choline metabolism and TMAO production have also been experimentally linked to infarct severity and adverse post-stroke outcomes, while clinical studies suggest that higher plasma TMAO levels may predict unfavorable outcomes in patients with acute ischemic stroke (Zhu et al., 2021; Zhai et al., 2019). Recent clinical evidence further indicates that circulating LPS burden changes after acute ischemic stroke and may be associated with neurological outcome, supporting the relevance of microbial translocation and barrier-sensitive inflammation (Błaż et al., 2024).

Direct evidence is less mature for CSVD and ICH. Cross-sectional human studies suggest associations between gut microbial composition and imaging-defined CSVD burden, cognitive or neuroimaging markers, and candidate microbial biomarkers; however, these studies remain limited by sample size, residual confounding, and lack of longitudinal repeated MRI–microbiome sampling (Saji et al., 2021; Fongang et al., 2023; Shi et al., 2023). Mendelian randomization analyses provide suggestive directional evidence consistent with possible causal relationships between gut microbiota and CSVD-related neuroimaging subtypes; however, these findings require replication in independent datasets and experimental validation and should not be interpreted as established causality (Huang et al., 2024). In ICH, early clinical and experimental studies suggest that gut dysbiosis, altered microbial diversity, and specific taxa such as Enterococcus or Lachnospiraceae may relate to neurological prognosis, but the evidence base remains much smaller than that for ischemic stroke and should not be treated as equivalent (Yu et al., 2021; Wang et al., 2024a; Jiang et al., 2024). Metabolite-focused clinical evidence also suggests that higher plasma TMAO levels may be associated with poor outcome after ICH, although larger validation cohorts are still needed (Zhai et al., 2021).

Beyond bacteria, multi-kingdom microbial signals are emerging as an important but lower-evidence domain. The gut virome, including bacteriophages, may reshape bacterial community structure and microbial metabolic capacity, while fungal communities may influence host immune thresholds through pattern-recognition pathways. Early virome-focused studies suggest that stroke is associated with altered gut viral composition and that focal cerebral ischemia can change the gut virome in experimental models (Wang et al., 2021; Chelluboina et al., 2022). By contrast, cerebrovascular mycobiome research remains largely inferential, although broader immunological literature indicates that commensal fungi can interact with host immune pathways in biologically meaningful ways (Underhill and Iliev, 2014). These domains move cerebrovascular microbiome research beyond bacterial taxonomic description toward microbial ecosystem dynamics, inter-kingdom interactions, and functional host–microbe mechanisms.

From the perspective of vertebrate digestive-system microbiology, the relevant unit of analysis is not a generic gut–brain axis but the intestinal microbial ecosystem itself, including strain-level composition, trophic interactions, functional genes, ecological resilience, and metabolic output. Representative colonic butyrate producers, including *Faecalibacterium prausnitzii*, *Roseburia* spp., and selected *Coprococcus* spp., may use different terminal pathways for butyrate synthesis, including the butyryl-CoA:acetate CoA-transferase and butyrate kinase pathways encoded by *but* and *buk*, respectively. Conversely, microbial trimethylamine production is more directly represented by functional genes such as *cutC/cutD* for anaerobic choline utilization and *cntA/cntB* for carnitine utilization than by broad phylum- or genus-level shifts. These examples illustrate why taxonomic abundance cannot be equated directly with microbial metabolic capacity. Accordingly, this review treats intestinal microorganisms as members of functional guilds and emphasizes gene content, cross-feeding, substrate utilization, and metabolite production as digestive-system microbial properties that may subsequently influence host neurovascular biology (Duncan et al., 2002; Louis et al., 2004; Vital et al., 2014; Rath et al., 2017).

A microbiology-centered synthesis is therefore needed. Many existing reviews describe the gut–brain axis in stroke or cerebrovascular disease broadly, but fewer distinguish microbial ecology, microbial metabolites, epithelial barrier injury, microbial translocation, immune programming, and causal inference across CSVD, ischemic stroke, and ICH. This distinction is essential because microbiome findings in cerebrovascular disease are strongly influenced by diet, antibiotics, constipation, hospitalization, enteral feeding, infection, vascular medications, frailty, and sampling time after stroke onset. Translational interpretation also requires caution: microbiome-based diagnostics, probiotics, prebiotics, postbiotics, fecal microbiota transplantation, metabolite-targeted strategies, and phage-based approaches remain promising but incompletely validated in cerebrovascular populations (Raghani et al., 2024; Gilbert et al., 2025).

**Figure 1:**
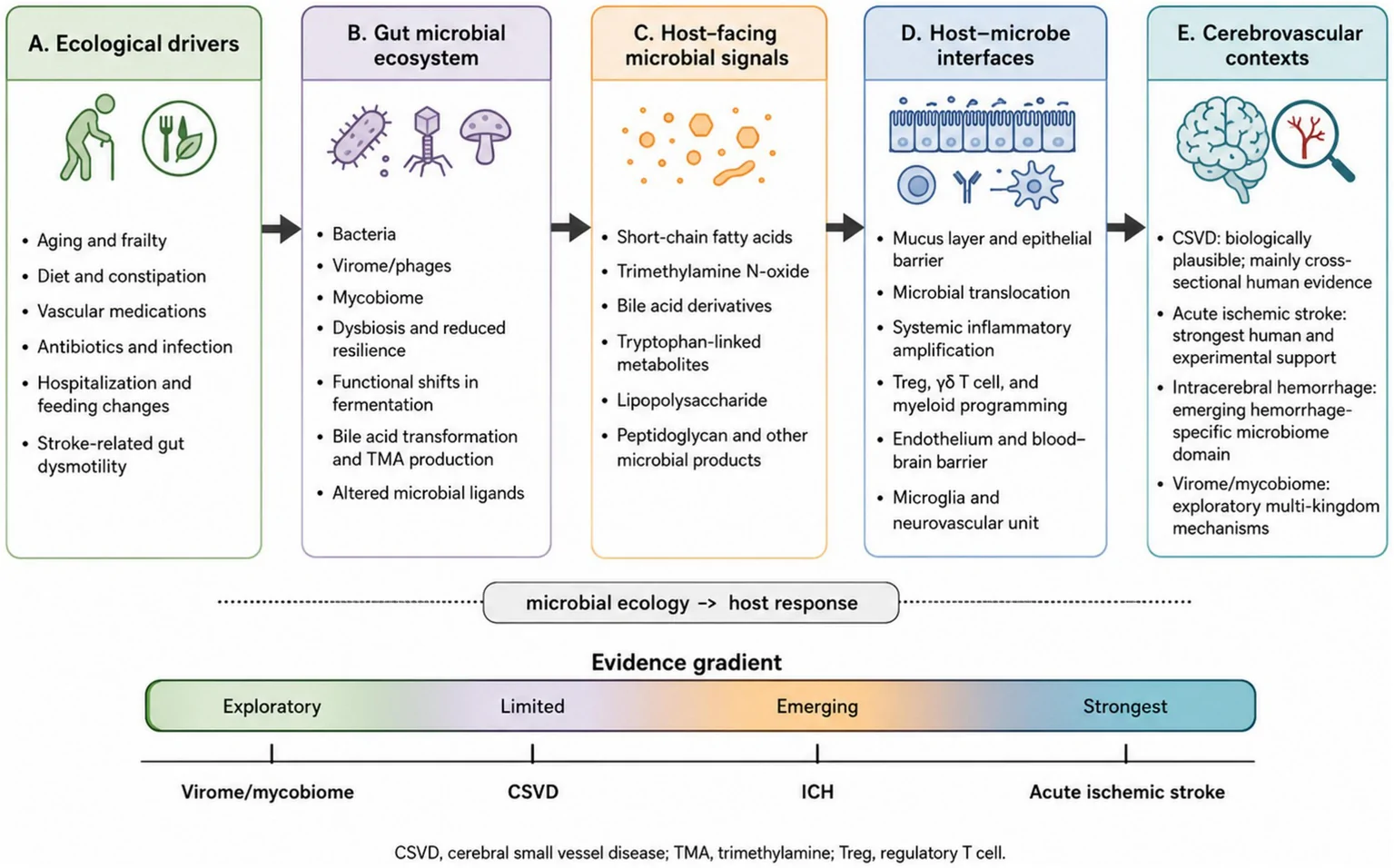
Microbiology-centered framework linking gut microbial ecosystems to cerebrovascular disease. Aging, frailty, diet, constipation, vascular medication exposure, antibiotics, infection, hospitalization, enteral feeding, and stroke-related gut dysmotility may reshape the intestinal microbial ecosystem by reducing microbial resilience and altering bacterial, viral, and fungal community structure. Dysbiotic ecosystems may modify host-facing microbial signals, including short-chain fatty acids (SCFAs), trimethylamine N-oxide (TMAO), bile acid derivatives, tryptophan-linked metabolites, lipopolysaccharide (LPS), peptidoglycan, and other microbial structural products. These signals interact with host–microbe interfaces, including the mucus layer, intestinal epithelial barrier, microbial translocation pathways, mucosal and systemic immune programming, endothelial activation, the blood–brain barrier, microglia, and the neurovascular unit. The strength of current evidence differs across cerebrovascular contexts. Acute ischemic stroke has the strongest human and experimental support, cerebral small vessel disease remains biologically plausible but is supported mainly by cross-sectional human evidence, intracerebral hemorrhage represents an emerging hemorrhage-specific microbiome domain, and virome/mycobiome mechanisms remain exploratory. The framework is intended to summarize candidate microbiome–immune–metabolic pathways and evidence maturity rather than to imply a single validated causal sequence across all cerebrovascular diseases. The evidence gradient reflects the qualitative criteria defined in Section 3 and indicates relative maturity within the reviewed cerebrovascular literature rather than formal GRADE certainty.

This review examines gut microbiome–immune–metabolic mechanisms across CSVD, acute ischemic stroke, and ICH with attention to differences in disease phenotype, sampling time, human cohort evidence, and experimental support. We prioritize direct human cerebrovascular evidence when available, use experimental studies to assess mechanistic plausibility, and clearly separate established, emerging, and hypothesis-generating domains. By reframing cerebrovascular disease through the structure, dynamics, and function of gut microbial ecosystems, this review aims to clarify how host–microbe interactions may shape neurovascular vulnerability, secondary injury, and recovery in aging. In addition to organizing mechanistic evidence, this review evaluates the translational readiness of candidate microbiome-directed interventions. We distinguish ecological approaches, live microbial interventions, postbiotics, fecal microbiota transplantation, defined microbial consortia, metabolite-targeted strategies, and phage-based approaches according to their biological targets, current evidence maturity, safety considerations, and potential disease-specific applications. Particular attention is given to the need for different intervention windows and clinical endpoints in CSVD, acute ischemic stroke, and ICH. The microbiology-centered framework of this review is summarized in Figure 1.

## 2 Review design and evidence framework

This article is a structured narrative review rather than a systematic review. PubMed/MEDLINE, Embase, and Web of Science were searched for studies published between January 1, 2010 and January 31, 2026 using combinations of terms related to the gut microbiome, dysbiosis, microbial metabolites, intestinal barrier dysfunction, virome, mycobiome, cerebral small vessel disease, white matter hyperintensities, lacunes, cerebral microbleeds, ischemic stroke, and intracerebral hemorrhage. Additional terms included blood–brain barrier, neuroinflammation, immune trafficking, lipopolysaccharide, short-chain fatty acids, trimethylamine N-oxide, bile acids, and tryptophan metabolism. A systematic review or meta-analysis was not attempted because the available literature spans heterogeneous disease phenotypes, sampling windows, sequencing platforms, metabolomic assays, and experimental models. Accordingly, no formal PRISMA flow diagram, risk-of-bias scoring, or quantitative evidence pooling was performed; the aim was to provide an evidence-organizing narrative synthesis rather than an exhaustive systematic review.

To supplement the translational analysis, ClinicalTrials.gov was searched on 24 July 2026 for registered probiotic, fecal microbiota transplantation, and other microbiome-directed intervention studies in stroke populations. Registry records were reviewed for study design, population, intervention, outcomes, recruitment status, and availability of posted results. Registered trials were used to describe the current translational landscape and were not interpreted as evidence of clinical efficacy in the absence of posted results.

Priority was given to direct human cerebrovascular evidence when available, especially studies using imaging-defined CSVD phenotypes or well-characterized acute stroke and ICH cohorts. Experimental studies, microbiota perturbation models, germ-free or antibiotic-treated models, metabolite-focused interventions, and adjacent host–microbe mechanistic studies were used to assess biological plausibility. Evidence was interpreted by separating human cerebrovascular findings from experimental perturbation data, adjacent mechanistic biology, and claims with immediate translational implications. We used a four-level qualitative evidence-maturity scheme based on four dimensions: (1) the amount and consistency of direct human cerebrovascular evidence; (2) the availability of longitudinal or repeated-sampling data; (3) the presence of functional microbial, metabolomic, barrier, immune, or neurovascular readouts; and (4) alignment with disease-specific experimental perturbation studies. “Exploratory” evidence was assigned when findings were derived mainly from isolated, small, adjacent-field, or multi-kingdom studies without independent replication or disease-specific perturbation. “Limited” evidence indicated more than one relevant human association but predominantly cross-sectional designs, substantial heterogeneity, and little functional or experimental validation. “Emerging” evidence indicated convergent human and disease-specific experimental findings, with at least some functional, longitudinal, or mechanistic linkage, but insufficient replication or intervention evidence. “Strongest” denoted the most mature evidence within the cerebrovascular conditions reviewed, characterized by multiple human cohorts, aligned functional readouts, and disease-specific experimental perturbation. These categories represent relative evidence maturity within this narrative review and should not be interpreted as formal GRADE ratings, proof of causality, or clinical-readiness classifications. The key primary and methodologically relevant studies informing this evidence organization are summarized in Supplementary Table 1, which distinguishes human observational evidence, genetic-instrument analyses, experimental perturbation studies, adjacent mechanistic biology, multi-kingdom exploratory evidence, and methodological or translational guidance. Because microbiome findings are highly sensitive to diet, antibiotics, hospitalization, infection, medication exposure, sample timing, sequencing platform, and analytical pipeline, these factors were considered when interpreting disease-specific evidence (Knight et al., 2018; Mirzayi et al., 2021; Gilbert et al., 2025).

## 3 Gut microbial ecology in aging and cerebrovascular vulnerability

Aging alters the ecological stability of the intestinal microbiome. In older hosts, microbial communities may show reduced resilience, altered taxonomic composition, impaired recovery after perturbation, and functional shifts affecting mucin utilization, SCFA production, bile acid transformation, and inflammatory ligand exposure. These ecological changes are relevant to cerebrovascular disease because CSVD, ischemic stroke, and ICH occur predominantly in older adults and often develop on a background of frailty, metabolic disease, vascular medication exposure, constipation, altered diet, and intermittent antibiotic use. More broadly, large-scale population microbiome research has shown that host and environmental factors, including medication use, diet, and health status, strongly shape gut microbial composition, reinforcing the need to control these exposures in cerebrovascular microbiome studies (Gacesa et al., 2022).

An ecology-centered interpretation also requires separation of taxonomic abundance from functional capacity. Butyrate production is distributed across phylogenetically diverse intestinal anaerobes and may proceed through different terminal pathways, principally those encoded by *but* or *buk*. Consequently, depletion of a single taxon does not necessarily indicate loss of total butyrate-producing capacity, because functionally related organisms may provide partial ecological redundancy. Conversely, simultaneous disruption of multiple members of a functional guild may reduce metabolite output even when overall community diversity changes only modestly. Microbial cross-feeding is also relevant: acetate or lactate generated by primary fermenters may be used by secondary fermenters for butyrate production. Shotgun metagenomics, metatranscriptomics, and targeted quantification of functional genes should therefore complement 16S rRNA gene profiling when evaluating microbial metabolic resilience in aging and cerebrovascular disease (Duncan et al., 2002; Louis et al., 2004; Vital et al., 2014).

Experimental evidence supports the biological relevance of age-associated microbial remodeling. Aged microbiota transfer can promote systemic inflammatory features in germ-free recipients, suggesting that age-related dysbiosis may actively contribute to inflammaging rather than merely reflect chronological aging (Fransen et al., 2017). Gut microorganisms also influence BBB integrity and microglial maturation, providing a plausible connection between intestinal microbial ecology and central nervous system immune–barrier function (Braniste et al., 2014; Erny et al., 2015). These studies do not prove that aging-related dysbiosis causes human cerebrovascular disease, but they support a framework in which microbial instability, epithelial barrier dysfunction, and systemic immune priming may lower neurovascular resilience.

In CSVD, this framework is most relevant to slow neurovascular vulnerability. White matter hyperintensities, lacunes, cerebral microbleeds, and enlarged perivascular spaces reflect partially overlapping but biologically non-identical processes (Wardlaw et al., 2013; Duering et al., 2023). Therefore, gut microbial signatures should not be interpreted as equivalent across all CSVD phenotypes. For acute ischemic stroke and ICH, microbial ecology may be relevant both before and after the event: premorbid microbial states may influence inflammatory set points, whereas acute brain injury, hospitalization, antibiotics, enteral feeding, infection, and altered gut motility may rapidly reshape the intestinal ecosystem (Kumar et al., 2024; Westendorp et al., 2022).

## 4 Disease-specific microbial signatures across CSVD, ischemic stroke, and ICH

Table 1 provides a compact, phrase-based evidence map across CSVD, acute ischemic stroke, ICH, shared acute cerebrovascular mechanisms, and multi-kingdom microbial domains. Sections 5.1–5.3 retain the detailed narrative interpretation, whereas Supplementary Table 1 provides study-level information on design, model, microbial or host-response focus, principal findings, contribution to the review framework, and evidence category. Disease-associated taxa should be interpreted as members of candidate functional guilds rather than as uniformly beneficial or harmful organisms, because strain-level gene content, substrate availability, microbial interactions, and treatment exposure may alter their metabolic output. Accordingly, genus-level associations reported below are treated as ecological or biomarker signals unless supported by strain-resolved sequencing, functional-gene analysis, metabolite measurements, or experimental perturbation.

**Table 1:** Disease-specific microbiome evidence across cerebrovascular conditions.

Cerebrovascular domain	Main human microbiome evidence	Main experimental or mechanistic support	Microbiology-centered interpretation	Major limitations	Evidence level in this review
Cerebral small vessel disease (CSVD)	Cross-sectional associations with CSVD burden, white matter hyperintensities, cognition, and candidate taxa; Mendelian randomization provides suggestive directional evidence requiring independent replication and experimental validation (Saji et al., 2021; Fongang et al., 2023; Shi et al., 2023; Huang et al., 2024).	Adjacent aging, BBB, endothelial, microglial, and inflammaging biology; few CSVD-specific perturbation models (Braniste et al., 2014; Erny et al., 2015; Fransen et al., 2017).	Plausible aging-microbiome-neurovascular interface; direct disease-specific causality unproven.	Small cross-sectional cohorts; heterogeneous imaging definitions; sparse repeated MRI-microbiome sampling; incomplete control of diet, medication, constipation, frailty, and cardiometabolic disease.	Limited: sparse human evidence; mainly adjacent aging/barrier biology.
Acute ischemic stroke	Multiple cohorts: dysbiosis, reduced SCFA-related signatures, circulating LPS, TMAO, stroke severity, and functional outcome associations (Xia et al., 2019; Tan et al., 2021; Zhai et al., 2019; Błaż et al., 2024).	Perturbation models: intestinal immune programming, γδ T cells, regulatory T cells, immune trafficking, barrier disruption, and stroke-induced dysbiosis (Benakis et al., 2016; Singh et al., 2016; Xu et al., 2021).	Most mature cerebrovascular context; bidirectional brain-gut disturbance with aligned human and experimental evidence.	Strong timing and treatment confounding; antibiotics, infection, dysphagia, enteral feeding, severity, gut dysmotility, and absent pre-stroke baselines.	Strongest within this review: most developed human-experimental alignment.
Intracerebral hemorrhage (ICH)	Small clinical cohorts: dysbiosis, diversity changes, and selected taxa associated with neurological recovery; plasma TMAO associated with poor outcome (Zhai et al., 2021; Wang et al., 2024a; Jiang et al., 2024).	ICH models: microbiota-dependent neuroinflammation; candidate links to NLRP3 signaling, white matter injury, hematoma resolution, and regulatory T-cell differentiation (Yu et al., 2021; Xiao et al., 2022; Li et al., 2023; Zheng et al., 2025).	Emerging hemorrhage-specific microbiome domain; biologically distinct from ischemic stroke.	Small cohorts; limited longitudinal sampling; sparse replication and perturbation-based validation.	Emerging hemorrhage-specific clinical and experimental evidence.
Shared acute cerebrovascular injury mechanisms	Human evidence dominated by ischemic stroke: dysbiosis, metabolites, permeability, microbial translocation, immune activation, and outcome associations; limited ICH replication (Tan et al., 2021; Błaż et al., 2024; Wang et al., 2024a).	Barrier disruption, microbiota-dependent immune trafficking, neuroinflammatory amplification, and BBB-related signaling after acute brain injury (Benakis et al., 2016; Singh et al., 2016; Xu et al., 2021).	Shared host-microbe interfaces, but disease-specific weighting and timing; not a single validated causal pathway.	Critical illness, antibiotics, infection, feeding route, gut dysmotility, immune suppression, and disease heterogeneity.	Emerging shared mechanisms; strongest in ischemic stroke.
Virome, phage, mycobiome, and other multi-kingdom domains	Early stroke-associated virome and phage-bacteria signals; direct cerebrovascular mycobiome evidence minimal (Wang et al., 2021).	Ischemia-induced virome remodeling in mice; adjacent fungal-immune biology (Chelluboina et al., 2022; Underhill and Iliev, 2014).	Exploratory ecosystem-level signals affecting bacterial fitness, gene transfer, metabolic capacity, and immune thresholds.	Small studies; sparse longitudinal profiling; limited perturbation; mycobiome evidence largely extrapolated from adjacent fields.	Exploratory multi-kingdom evidence.

Evidence levels follow the qualitative evidence-maturity criteria defined in Section 3. They indicate relative maturity within this review and should not be interpreted as formal GRADE ratings or proof of causality. BBB, blood–brain barrier; CSVD, cerebral small vessel disease; ICH, intracerebral hemorrhage; LPS, lipopolysaccharide; MRI, magnetic resonance imaging; SCFAs, short-chain fatty acids; TMAO, trimethylamine N-oxide.

### 4.1 Cerebral small vessel disease

Human CSVD microbiome studies are still sparse and are constrained mainly by cross-sectional imaging, small sample sizes, and the absence of repeated MRI–microbiome sampling. Adjacent MRI-based metabolomic studies further suggest that circulating metabolic signatures are associated with white matter hyperintensity burden and other brain MRI markers in older adults, although these studies do not establish gut microbiome specificity or causality (Li et al., 2019; Sliz et al., 2022; Wang et al., 2025). Cross-sectional studies suggest that gut microbial composition may be associated with total CSVD burden, white matter hyperintensity load, cognitive status, or candidate imaging markers (Saji et al., 2021; Fongang et al., 2023; Shi et al., 2023). A bidirectional Mendelian randomization study provided suggestive directional evidence consistent with possible causal relationships between selected gut microbial taxa and CSVD-related imaging subtypes. However, these genetic-instrument findings require replication in independent datasets and experimental validation and should not be interpreted as established causality or mechanistic proof (Huang et al., 2024). Among CSVD phenotypes, white matter hyperintensity burden currently provides the most accessible imaging readout for microbiome-associated analyses. Evidence for lacunes, cerebral microbleeds, and enlarged perivascular spaces remains more fragmented because most available studies do not provide phenotype-specific microbial associations. Future CSVD microbiome studies should therefore report individual imaging markers separately rather than treating total CSVD burden as a uniform endpoint.

A cautious interpretation is that CSVD should be framed as a microbiome–aging–neurovascular interface rather than as a fully validated microbiome-driven disease. Current CSVD studies are constrained by cross-sectional design, limited sample size, heterogeneous imaging definitions, incomplete dietary and medication adjustment, and lack of repeated stool, plasma metabolite, and MRI sampling (Saji et al., 2021; Fongang et al., 2023; Shi et al., 2023). Future CSVD microbiome research should separate WMH, lacunes, microbleeds, and enlarged perivascular spaces; integrate shotgun metagenomics and metabolomics; and determine whether microbial functional pathways track imaging progression over time (Wardlaw et al., 2013; Duering et al., 2023; Knight et al., 2018).

### 4.2 Acute ischemic stroke

Acute ischemic stroke is the best-characterized cerebrovascular setting for studying rapid brain–gut–microbiome coupling because injury onset can be temporally defined and early inflammatory, barrier-related, and metabolic changes can be sampled over hours to days. Experimental studies have shown that commensal microbiota influence ischemic stroke outcome through intestinal immune programming, including effects on regulatory T cells, IL-17-positive γδ T cells, and immune-cell trafficking toward the injured brain (Benakis et al., 2016; Singh et al., 2016). Stroke can also rapidly induce gut dysbiosis, including expansion of potentially pro-inflammatory taxa such as Enterobacteriaceae, and this dysbiosis may exacerbate brain infarction (Xu et al., 2021).

Human studies support the clinical relevance of these observations. Earlier clinical studies showed that gut dysbiosis in ischemic stroke is associated with host metabolism and systemic inflammation, while more recent oral–gut microbiome and Mendelian randomization studies suggest potential links between microbial signatures and functional outcome after ischemic stroke (Yamashiro et al., 2017; Liang et al., 2025; Qu et al., 2024). Gut dysbiosis signatures have been associated with stroke severity, brain injury, and prognosis (Xia et al., 2019). Reduced SCFA-producing bacteria and lower fecal SCFA concentrations have been associated with poor functional outcome after acute ischemic stroke (Tan et al., 2021). Acute stroke can also alter gut motility and enteric neuronal function, providing a physiological route through which cerebral injury may rapidly disturb the intestinal ecosystem (Kumar et al., 2024). Microbiome disruption may also intersect with post-stroke complications, including gastrointestinal dysfunction, infection, cognitive impairment, depression, and hemorrhagic transformation, although the available evidence remains predominantly associative or mechanistically inferential (Zhang et al., 2024). These findings suggest that acute ischemic stroke is not only a brain-to-gut perturbation but also a context in which altered microbial ecology may feed back into immune activation, barrier dysfunction, and neurological recovery.

### 4.3 Intracerebral hemorrhage

ICH should be analyzed separately from ischemic stroke because hemorrhagic injury involves hematoma expansion, blood degradation products, edema, inflammasome activation, and hematoma clearance rather than arterial occlusion and reperfusion alone. Direct clinical evidence remains limited but is increasing. Studies suggest that ICH is associated with gut microbiota dysbiosis and that microbial diversity or specific taxa may relate to neurological recovery (Wang et al., 2024a; Jiang et al., 2024). Experimental work further suggests that ICH-induced gut dysbiosis may aggravate neuroinflammation (Yu et al., 2021). Additional experimental studies have linked gut microbiota-related pathways with NLRP3 inflammasome signaling, white matter injury, hematoma resolution, and Treg differentiation after ICH, supporting the need for hemorrhage-specific microbiome models rather than direct extrapolation from ischemic stroke (Xiao et al., 2022; Li et al., 2023; Zheng et al., 2025).

Metabolite-focused evidence also supports a cautious microbiome–ICH connection. Higher plasma TMAO levels have been associated with poor outcome after ICH (Zhai et al., 2021). The main challenge for ICH microbiome research is therefore not only cohort size, but also the need to link gut microbial changes to hemorrhage-specific processes such as perihematomal inflammation, edema formation, white matter injury, and hematoma resolution.

## 5 Microbial metabolites and structural products as host effectors

Table 2 provides a compact source–pathway–target–evidence map for the major microbial metabolites and structural products considered in this review. The principal host-facing signals discussed below include SCFAs, TMAO, LPS, bile acid derivatives, tryptophan-linked metabolites, peptidoglycan, and multi-kingdom microbial products. The narrative focuses on mechanistic interpretation, functional specificity, and evidence boundaries rather than repeating the table entries. Microbial metabolites provide a functional bridge between gut community structure and host cerebrovascular biology. Taxonomic dysbiosis alone is insufficient to explain disease mechanisms because different microbial communities may converge on similar metabolic outputs, and the same taxon may have different functions depending on ecological context. Therefore, microbial metabolites and structural products should be treated as central host effectors in cerebrovascular microbiome research (Knight et al., 2018; Gilbert et al., 2025). Recent reviews of gut microbiota-derived metabolites also emphasize that SCFAs, TMAO, bile acid derivatives, and tryptophan-linked metabolites may act as host-facing biochemical mediators within the gut–brain axis, but their effects depend on microbial source, host context, and disease timing (Peh et al., 2022; Ahmed et al., 2022; Zhang et al., 2023; Qin et al., 2025).

**Table 2:** Microbial metabolites and structural products relevant to cerebrovascular disease.

Microbial signal or product	Main microbial source or pathway	Candidate host targets	Cerebrovascular relevance	Current interpretation
Short-chain fatty acids (SCFAs)	Fiber-fermenting anaerobes, including *Faecalibacterium prausnitzii*, *Roseburia* spp., and selected *Coprococcus* spp.; acetate, propionate, and *but*yrate; terminal *but*yrate pathways involving *but* or *buk* (Duncan et al., 2002; Louis et al., 2004; Vital et al., 2014).	Epithelium and mucus; regulatory T cells and myeloid cells; endothelium and BBB; microglia.	Reduced SCFA-producing bacteria and lower fecal SCFAs associated with greater acute ischemic stroke severity and poorer functional outcome (Tan et al., 2021).	Plausibly protective; timing-, dose-, and context-dependent; limited direct evidence in CSVD and ICH.
Trimethylamine N-oxide (TMAO)	Dietary choline/carnitine - > microbial TMA via *cutC/cutD*, *cntA/cntB*, or related pathways - > hepatic TMAO (Rath et al., 2017).	Platelets; endothelium; thrombosis and inflammatory pathways; systemic metabolism.	Experimental stroke-severity evidence and human prognostic associations in acute ischemic stroke and ICH (Zhu et al., 2021; Zhai et al., 2019, 2021).	Relatively mature candidate pathway; biomarker and possible mediator, not a universal causal factor.
Lipopolysaccharide (LPS)	Gram-negative bacterial outer-membrane component; systemic exposure increased by barrier injury and microbial translocation.	Innate immune receptors; monocytes; endothelium; systemic inflammation; BBB.	Post-stroke circulating LPS changes associated with acute ischemic stroke outcome (Błaż et al., 2024).	Barrier/translocation marker; inflammatory plausibility supported; disease-specific causality unproven.
Bile acid derivatives	Host-derived primary bile acids; microbial deconjugation, dehydroxylation, and secondary bile acid formation.	FXR and TGR5; systemic metabolism; endothelium; immune cells.	Microbiota-related bile acid profiles associated with acute ischemic stroke prognosis (Wang et al., 2024b).	Emerging pathway; effects may depend on bile acid subtype, timing, and host metabolic state.
Tryptophan-linked metabolites	Microbial indole derivatives and host kynurenine/serotonin pathways; aryl hydrocarbon receptor-linked signaling.	Intestinal barrier; aryl hydrocarbon receptor; immune and glial cells; endothelium.	Candidate biomarkers of acute ischemic stroke severity and prognosis (Pan et al., 2025; Qin et al., 2025).	Early evidence; microbial versus host source requires integrated microbiome-metabolome analysis.
Peptidoglycan and other bacterial structural products	Bacterial cell-wall turnover; increased exposure with dysbiosis, barrier failure, or microbial translocation.	Innate immune sensors; monocytes/macrophages; endothelium; systemic inflammatory pathways; neurovascular unit.	Candidate contri*but*ors to innate immune activation after acute brain injury; direct human cerebrovascular evidence sparse.	Hypothesis-generating host-microbe interface; lower evidence than LPS, SCFAs, and TMAO.
Phage-associated and virome-related signals	Bacteriophages and enteric viruses; effects on bacterial fitness, gene transfer, community structure, and microbial functional potential.	Bacterial ecology; microbial metabolism; immune-ligand exposure; mucosal immune tone.	Altered stroke-associated virome in humans; ischemia-induced virome remodeling in mice (Wang et al., 2021; Chelluboina et al., 2022).	Exploratory ecological relevance; disease-specific causality and therapeutic utility unproven.
Fungal and mycobiome-related signals	Gut fungi, fungal cell-wall ligands, and bacterial-fungal ecological interactions.	Mucosal immunity; innate immune receptors; inflammatory thresholds; inter-kingdom ecology.	Direct cerebrovascular evidence sparse; support derived mainly from adjacent fungal-immune biology (Underhill and Iliev, 2014).	Highly exploratory; appropriate for hypothesis-driven multi-kingdom profiling.

Taxonomic associations do not necessarily indicate pathway activity. Functional interpretation should preferentially incorporate strain-resolved sequencing, microbial-gene analysis, metabolite measurements, or experimental perturbation. AhR, aryl hydrocarbon receptor; BBB, blood–brain barrier; CSVD, cerebral small vessel disease; FXR, farnesoid X receptor; ICH, intracerebral hemorrhage; LPS, lipopolysaccharide; SCFAs, short-chain fatty acids; TGR5, Takeda G protein-coupled receptor 5; TMA, trimethylamine; TMAO, trimethylamine N-oxide.

SCFAs are among the most studied microbial metabolites in stroke. Acetate, propionate, and butyrate can influence epithelial barrier integrity, regulatory T-cell differentiation, myeloid activation, endothelial function, and microglial responses. In acute ischemic stroke, reduced SCFA-producing bacteria and decreased fecal SCFA concentrations have been linked to unfavorable functional outcomes (Tan et al., 2021). These associations support a protective hypothesis, although timing, dose, microbial source, and host inflammatory state may influence the direction and magnitude of effect.

TMAO represents a potentially adverse microbial–metabolic pathway in cerebrovascular disease. Gut microbial metabolism of choline, carnitine, and related nutrients generates trimethylamine (TMA), which is converted by hepatic flavin monooxygenases into TMAO. At the microbial level, TMA-generating capacity is encoded by several substrate-specific pathways. The *cutC/cutD* system mediates the anaerobic conversion of choline to TMA, whereas *cntA/cntB* and related *yeaW/yeaX* systems contribute to carnitine-derived TMA formation. These genes are distributed discontinuously across phylogenetically diverse intestinal bacteria and may occur at relatively low abundance. Therefore, genus-level taxonomic profiles cannot reliably establish TMA-producing capacity. Cerebrovascular studies should pair circulating TMAO measurements with shotgun metagenomics, metatranscriptomics, or targeted quantification of TMA-generating genes rather than infer pathway activity from broad compositional changes alone (Rath et al., 2017). Experimental and clinical studies suggest that this pathway may influence thrombosis, vascular inflammation, infarct severity, and post-stroke outcome (Zhu et al., 2021; Zhai et al., 2019). In ICH, elevated plasma TMAO has also been associated with poor prognosis (Zhai et al., 2021). However, TMAO should be interpreted as a context-sensitive marker and potential mediator rather than a single universal cerebrovascular toxin.

LPS and other microbial structural products link barrier dysfunction to systemic inflammatory amplification. After acute stroke, impaired intestinal motility, epithelial injury, altered mucus function, and dysbiosis may increase exposure to microbial products. Recent clinical evidence suggests that circulating LPS burden changes after acute ischemic stroke and may be associated with outcome (Błaż et al., 2024). This supports the relevance of microbial translocation and barrier-sensitive inflammation as host–microbe interfaces in acute cerebrovascular injury.

Bile acid derivatives and tryptophan-linked metabolites are additional microbial pathways with potential cerebrovascular relevance. Microbial bile acid transformation can influence farnesoid X receptor and TGR5 signaling, systemic metabolism, endothelial activation, and immune tone. Clinical evidence suggests that gut microbiota-related bile acid profiles may be associated with prognosis after ischemic stroke (Wang et al., 2024b). Tryptophan metabolites may signal through aryl hydrocarbon receptor-related pathways and influence barrier regulation, glial activation, and inflammatory responses. Recent metabolomics evidence also suggests that tryptophan metabolites may help predict acute ischemic stroke severity and prognosis (Pan et al., 2025). These pathways remain less established than TMAO or SCFAs in human stroke cohorts, but they are important targets for future metabolomics-integrated microbiome studies.

## 6 Host–microbe interfaces: intestinal barrier, immune programming, and neurovascular signaling

The gut–brain axis in cerebrovascular disease should be interpreted as a set of host–microbe interfaces rather than as a linear pathway. The first interface is the intestinal epithelial barrier. Dysbiosis, mucus layer disruption, altered tight junction regulation, impaired motility, and critical illness-related changes may increase microbial ligand exposure. This can amplify systemic inflammation and potentially affect endothelial and BBB function (Braniste et al., 2014; Błaż et al., 2024; Kumar et al., 2024).

Recent mechanistic studies further suggest that intestinal epithelial signaling and neuromodulatory pathways, including TLR4-sensitive intestinal signaling and vagus nerve stimulation-related barrier modulation, may influence post-stroke gut–brain injury responses (Huang et al., 2026; Wang Z. et al., 2024).

The second interface is immune programming. Gut microorganisms influence regulatory T cells, γδ T cells, myeloid cells, and cytokine networks. In ischemic stroke models, microbiota-dependent immune changes can affect neuroinflammation and infarct outcome (Benakis et al., 2016; Singh et al., 2016). This is especially important because acute stroke is accompanied by both inflammatory activation and immunodepression, which together influence secondary brain injury and infection susceptibility (Westendorp et al., 2022).

The third interface is the neurovascular unit. Microbial metabolites and inflammatory ligands may influence endothelial activation, BBB permeability, pericyte function, microglial priming, and leukocyte trafficking. Experimental evidence that gut microbiota regulate BBB integrity and microglial maturation supports this interface biologically (Braniste et al., 2014; Erny et al., 2015). However, direct human evidence linking specific microbial pathways to neurovascular unit injury remains limited, especially in CSVD and ICH.

## 7 Multi-kingdom microbial signals: virome, phage, and mycobiome

Most cerebrovascular microbiome studies focus on bacteria, but the intestinal ecosystem also includes bacteriophages, eukaryotic viruses, fungi, archaea, and microbial mobile genetic elements. These domains may influence bacterial community structure, metabolic potential, immune ligand exposure, and ecological resilience. Including these domains helps shift interpretation from bacterial taxonomy alone toward multi-kingdom ecosystem dynamics, microbial functional potential, and inter-kingdom immune signaling.

Early virome-focused studies suggest that stroke may be associated with altered gut viral composition and phage–bacteria interaction patterns (Wang et al., 2021). Experimental focal cerebral ischemia can also alter gut virome structure in mice (Chelluboina et al., 2022). However, cerebrovascular virome evidence remains small in scale and concentrated mainly in acute ischemic settings. Mycobiome evidence is even more indirect. Broader immunological literature shows that commensal fungi can interact with host immune pathways (Underhill and Iliev, 2014), but cerebrovascular mycobiome studies are not yet sufficiently developed to support strong disease-specific conclusions.

Although phage-based strategies have been proposed as future tools for stroke recovery, current evidence remains conceptual or preclinical and should not be presented as an established cerebrovascular intervention (Li et al., 2024).

## 8 Methodological limitations in cerebrovascular microbiome studies

Table 3 is presented as a concise operational checklist for future cerebrovascular microbiome studies, whereas the narrative below explains the methodological rationale and major sources of bias. Methodological rigor is central to cerebrovascular microbiome research. First, stool sampling time is critical. Samples obtained before stroke, within hours of onset, during hospitalization, after antibiotics, or during rehabilitation may reflect different biological states. Without time-resolved sampling, it is difficult to distinguish premorbid microbial risk factors from stroke-induced dysbiosis (Xu et al., 2021; Kumar et al., 2024; Mirzayi et al., 2021).

**Table 3:** Methodological checklist for future cerebrovascular microbiome studies.

Methodological domain	Recommended practice	Why it matters	Relevant cerebrovascular context
Study design	Use longitudinal cohorts with repeated stool, blood, imaging, and clinical sampling.	Distinguishes premorbid microbial states from disease-induced and treatment-related changes.	All; especially CSVD and recovery cohorts.
Sampling time	Prespecify onset-to-sampling intervals; sample before antibiotics when feasible and during recovery.	Captures rapid post-event shifts and treatment effects.	Acute ischemic stroke; ICH.
CSVD imaging assessment	Report white matter hyperintensities, lacunes, microbleeds, and perivascular spaces separately; repeat MRI when feasible.	CSVD markers are biologically non-equivalent.	CSVD.
Clinical confounder control	Record diet, constipation, frailty, disease severity, hospitalization, feeding route, and major comorbidities.	Reduces non-disease microbial signals and residual confounding.	All.
Antibiotic and infection exposure	Record agent, dose, duration, timing, infection site, and inflammatory markers; stratify or perform sensitivity analyses.	Antibiotics and infection rapidly remodel microbiota and metabolites.	Acute ischemic stroke; ICH.
Diet and nutritional status	Assess habitual diet, fiber intake, dysphagia, enteral feeding, malnutrition, and in-hospital dietary change.	Directly affects SCFAs, bile acids, TMA/TMAO, and ecological resilience.	All.
Medication exposure	Record statins, antiplatelets, anticoagulants, antihypertensives, antidiabetic drugs, and proton pump inhibitors.	Medication effects may mimic disease-associated microbiome signals.	All.
Sequencing strategy	Report extraction, platform, target region, database, and pipeline; add shotgun metagenomics when feasible.	Improves species-, strain-, gene-, and pathway-level resolution.	All.
Functional profiling	Quantify SCFA, TMA, bile acid, tryptophan, and inflammatory-ligand pathways; include targeted *but*, *buk*, *cutC*, and *cntA* assays when relevant.	Taxonomic abundance does not establish microbial metabolic capacity.	All.
Metabolomics integration	Pair fecal and plasma metabolomics with microbiome profiling.	Links microbial function with host-facing biochemical exposure.	All; especially acute ischemic stroke.
Host immune profiling	Measure cytokines, immune-cell phenotypes, endothelial markers, and barrier-related biomarkers.	Connects microbial signals with immune and neurovascular responses.	Acute ischemic stroke; ICH.
Barrier function assessment	Assess permeability and translocation using complementary LPS/endotoxin, tight-junction, and inflammatory readouts.	Tests the barrier-mediated host-microbe interface.	Acute ischemic stroke; ICH.
Multi-kingdom profiling	Profile virome, phages, and mycobiome in hypothesis-driven cohorts or nested subsets.	Captures inter-kingdom ecology beyond bacterial taxonomy.	Exploratory; all domains.
Causal validation	Use germ-free models, microbial depletion, fecal microbiota transfer, defined consortia, metabolite add-back, and receptor-specific perturbation.	Tests whether microbial pathways modify injury rather than merely correlate with it.	All; especially acute ischemic stroke and ICH models.
External validation	Replicate signatures and prediction models across cohorts, populations, and analytical pipelines.	Addresses geography, diet, batch, platform, and clinical-setting dependence.	All.
Data reporting and reproducibility	Follow STORMS-compatible reporting for collection, storage, extraction, analysis, covariates, and data access.	Enables reproducibility, comparison, and secondary analysis.	All.
Translational endpoint selection	Link microbiome measures to neurological, imaging, cognitive, infectious, survival, and quality-of-life outcomes.	Prevents taxonomic change from being misinterpreted as clinical benefit.	All.
Intervention characterization	Report strain or consortium identity, formulation, viability, dose, route, duration, storage, adherence, and concomitant exposures.	Intervention effects are product-, host-, and context-specific.	All; especially acute ischemic stroke and ICH intervention studies.
Target engagement and safety	Confirm the intended functional, metabolite, barrier, or immune effect; prospectively capture adverse events.	Taxonomic change alone does not demonstrate mechanism, benefit, or safety.	All; especially acute ischemic stroke and ICH.
Therapeutic interpretation	Describe microbiome-directed strategies as investigational unless supported by disease-specific clinical trials.	Association or preclinical efficacy does not establish clinical effectiveness.	All.

This table is intended as an operational checklist. Detailed methodological rationale and supporting evidence are provided in Section 9. AIS, acute ischemic stroke; CSVD, cerebral small vessel disease; ICH, intracerebral hemorrhage; LPS, lipopolysaccharide; MRI, magnetic resonance imaging; SCFAs, short-chain fatty acids; TMA, trimethylamine; TMAO, trimethylamine N-oxide.

Second, clinical confounding is substantial. Diet, antibiotics, proton pump inhibitors, statins, antihypertensive drugs, antidiabetic medications, constipation, dysphagia, enteral feeding, infection, immobility, frailty, and stroke severity can all influence the gut microbiome. These variables should be measured prospectively and included in sensitivity analyses (Gacesa et al., 2022; Knight et al., 2018; Mirzayi et al., 2021).

Third, sequencing and analytical methods differ across studies. 16S rRNA gene sequencing provides taxonomic profiling but limited strain-level and functional resolution. Shotgun metagenomics can identify species, strains, microbial genes, and functional potential but requires greater sequencing depth and careful bioinformatic control. Metabolomics is essential for linking microbial ecology to host signaling, but plasma, fecal, and cerebrospinal fluid metabolite profiles may represent different biological compartments (Knight et al., 2018; Gilbert et al., 2025).

Fourth, causality remains difficult to infer. Cross-sectional associations do not establish whether dysbiosis drives cerebrovascular injury, reflects disease severity, or results from clinical exposures. Stronger inference will require longitudinal cohorts, repeated MRI, paired stool and plasma sampling, germ-free or antibiotic-treated aged models, fecal microbiota transfer, defined consortia, metabolite add-back experiments, receptor-specific interventions, and standardized reporting of sample collection, storage, DNA extraction, sequencing, bioinformatics, covariate adjustment, and data availability (Knight et al., 2018; Mirzayi et al., 2021; Gilbert et al., 2025). Fifth, microbiome intervention studies require standardized characterization of the therapeutic product and biological target. Dietary interventions should report nutrient composition, fiber intake, major dietary changes, adherence, and background nutritional status. Probiotic and defined-consortium studies should specify strain identity, formulation, viability, dose, administration route, treatment duration, storage conditions, and concomitant antibiotic exposure. Postbiotic and metabolite-targeted studies should define the active component, dose, pharmacodynamic readout, and intended host or microbial pathway. Fecal microbiota transplantation studies require detailed donor screening, preparation and administration protocols, and longitudinal assessment of engraftment and adverse events. Across all intervention categories, changes in microbial taxonomy alone should not be considered evidence of therapeutic target engagement. Studies should demonstrate modification of the intended microbial function, metabolite, intestinal barrier marker, immune pathway, or host-response signal and connect these changes to disease-relevant clinical outcomes (Table 4).

**Table 4:** Candidate microbiome-directed therapeutic strategies and translational considerations in cerebrovascular disease.

Therapeutic strategy	Primary target or proposed mechanism	Current evidence stage	Potential cerebrovascular setting	Major concerns	Recommended translational endpoints
Dietary modification and prebiotics	Modify substrate availability, SCFA production, bile acid transformation, TMA/TMAO-related metabolism, and microbial ecological resilience.	Indirect human evidence and mechanistic support; limited disease-specific intervention evidence.	CSVD prevention, post-stroke recovery, and secondary prevention.	Dietary heterogeneity, adherence, malnutrition, dysphagia, enteral feeding, and baseline microbiome dependence.	Dietary adherence, microbial functional pathways, SCFAs, bile acids, TMAO, inflammatory markers, MRI progression, cognition, and functional outcome.
Probiotics	Introduce strain-specific barrier-, immune-, or metabolite-modifying functions.	Preclinical and early clinical-trial activity; no posted trial results or established cerebrovascular efficacy (NCT04954846; NCT05477732).	Acute ischemic stroke and stable post-stroke rehabilitation settings under clinical-trial evaluation.	Strain specificity, formulation, viability, antibiotic exposure, unstable engraftment, and host heterogeneity.	Strain detection, functional target engagement, barrier and immune markers, infection, tolerability, and neurological outcome.
Postbiotics	Deliver defined microbial products or inactivated microbial components without requiring live-organism engraftment.	Mechanistically plausible; direct cerebrovascular intervention evidence remains very limited.	Potentially acute or recovery-phase studies after safety evaluation.	Uncertain active components, dose, receptor specificity, and pharmacokinetics.	Active-component exposure, metabolite or receptor engagement, barrier markers, inflammation, safety, and functional outcome.
Fecal microbiota transplantation	Broad ecological transfer of microbial communities, metabolites, phages, fungi, and structural products.	Useful for experimental causal testing; insufficient clinical evidence.	No established cerebrovascular indication.	Donor variability, pathogen or resistance-gene transfer, administration route, engraftment uncertainty, antibiotics, dysphagia, and immune vulnerability.	Safety, engraftment, microbial functional change, metabolite profiles, infection, and disease-specific clinical outcomes.
Defined microbial consortia	Introduce selected organisms with specified metabolic or immune functions.	Preclinical and early translational concept; no established cerebrovascular clinical evidence.	Mechanism-focused prevention or recovery studies.	Community stability, manufacturing, strain interactions, host dependence, and ecological off-target effects.	Consortium persistence, functional gene and metabolite changes, safety, immune responses, and clinical outcomes.
SCFA-oriented approaches	Increase SCFA production or directly provide acetate, propionate, butyrate, or related compounds.	Human observational and mechanistic support; limited therapeutic evidence (Tan et al., 2021; Peh et al., 2022).	Mainly ischemic stroke recovery and prevention-oriented studies.	SCFA subtype, dose, route, timing, tissue exposure, and context-dependent effects.	Fecal and plasma SCFAs, barrier integrity, Treg and myeloid responses, infection, neurological recovery, and functional outcome.
TMA/TMAO-targeted approaches	Reduce microbial TMA production, modify dietary substrate exposure, or alter downstream host metabolism.	Relatively strong mechanistic and biomarker evidence; limited interventional evidence (Zhu et al., 2021; Zhai et al., 2019, 2021).	Ischemic stroke prevention or outcome studies; exploratory in ICH.	Diet, renal clearance, hepatic metabolism, comorbidity, and different implications in ischemic and hemorrhagic disease.	TMA/TMAO levels, microbial TMA pathways, platelet or endothelial markers, recurrent vascular events, neurological and functional outcomes.
Bile acid and tryptophan pathway modulation	Modify FXR, TGR5, AhR, barrier, metabolic, glial, and immune signaling.	Emerging human metabolomic and mechanistic evidence (Wang et al., 2024b; Pan et al., 2025; Qin et al., 2025).	Exploratory across cerebrovascular conditions.	Metabolite subtype, microbial versus host source, timing, and host metabolic state.	Individual bile acids, tryptophan metabolites, microbial pathways, receptor-related markers, inflammation, and clinical outcome.
Phage-based strategies	Selectively target bacterial strains or adverse microbial functions.	Conceptual or preclinical in cerebrovascular disease (Li et al., 2024).	No established clinical setting.	Narrow host range, resistance, immune clearance, gene transfer, dosing, and ecosystem-level off-target effects.	Target-bacterium reduction, phage persistence, microbial function, LPS-related exposure, ecological effects, safety, and clinical outcome.

Evidence-stage assignments are qualitative and reflect the human observational, experimental, interventional, and methodological literature synthesized in Sections 6 and 10. They are intended to indicate relative evidence maturity and should not be interpreted as formal GRADE ratings. AhR, aryl hydrocarbon receptor; CSVD, cerebral small vessel disease; FXR, farnesoid X receptor; ICH, intracerebral hemorrhage; LPS, lipopolysaccharide; SCFAs, short-chain fatty acids; TGR5, Takeda G protein-coupled receptor 5; TMA, trimethylamine; TMAO, trimethylamine N-oxide.

## 9 Therapeutic strategies, translational implications, and clinical boundaries

### 9.1 Translational framework and evidence hierarchy

Microbiome-directed treatment in cerebrovascular disease should not be considered a single therapeutic category. Dietary modification, prebiotics, probiotics, postbiotics, fecal microbiota transplantation, defined microbial consortia, metabolite-targeted interventions, and phage-based approaches differ substantially in biological specificity, standardization, safety, and translational readiness. Their effects are also likely to depend on disease phenotype, treatment timing, baseline diet, medication exposure, intestinal motility, infection status, and host immune competence. Therapeutic interpretation should therefore move beyond whether an intervention broadly “corrects dysbiosis” and instead determine which microbial function is modified, which host-facing signal is altered, and whether the resulting effect is beneficial within a defined cerebrovascular context.

The evidence supporting an intervention should also be distinguished from evidence supporting the biological pathway that it targets. A microbial taxon, metabolite, or dysbiosis index associated with stroke severity or prognosis may represent a biomarker, a consequence of acute illness, or a confounded clinical exposure rather than a therapeutically modifiable mediator. Likewise, improvement in an experimental microbiota-transfer or metabolite-add-back model does not establish safety or efficacy in patients. A staged translational pathway is therefore required, progressing from mechanistic identification and experimental perturbation to human target-engagement studies, safety and feasibility trials, and ultimately disease-specific randomized clinical evaluation. At present, no microbiome-directed intervention has sufficient evidence to support routine treatment of CSVD, acute ischemic stroke, or ICH (Gilbert et al., 2025). Because direct disease-specific intervention evidence remains sparse for several therapeutic categories, the following discussion focuses on translational rationale, safety boundaries, and trial-design requirements rather than established clinical efficacy.

### 9.2 Dietary interventions, prebiotics, probiotics, and postbiotics

Dietary interventions may alter microbial substrate availability, SCFA production, bile acid transformation, TMA/TMAO-related metabolism, intestinal barrier function, and microbial ecological resilience. Increasing dietary fiber may support microbial fermentation and SCFA production, whereas dietary choline, carnitine, fat composition, and protein sources may influence other microbial metabolic pathways. However, dietary effects are not exclusively microbiome-mediated, and the same dietary intervention may produce different microbial and metabolic responses according to baseline microbiome function, medication use, renal function, nutritional status, and adherence.

Long-term dietary interventions may be more suitable for prevention-oriented CSVD studies, secondary stroke prevention, and recovery-phase cohorts than for the hyperacute phase of cerebrovascular injury. In hospitalized stroke patients, dysphagia, enteral feeding, aspiration risk, reduced intestinal motility, infection, antibiotic treatment, and rapid changes in nutritional intake may substantially modify both feasibility and biological response. Dietary studies should therefore document habitual diet, major in-hospital dietary changes, fiber intake, feeding route, swallowing status, malnutrition, and adherence rather than attributing all post-intervention changes to the microbiome.

Prebiotics, probiotics, and postbiotics should also be considered separately. Prebiotics modify the substrates available to resident microbial communities and may be ineffective when the relevant functional organisms are absent. Probiotic effects are likely to be strain-specific and should not be generalized from bacterial genus or species names alone. Probiotic studies should report strain identity, dose, viability, formulation, storage, administration route, and concomitant antibiotic exposure. Postbiotics may offer advantages in standardization because they do not require stable colonization by live microorganisms, but their active components, doses, host receptors, and pharmacodynamic effects must be clearly defined. For all three approaches, evidence of increased microbial diversity or changes in selected taxa should be accompanied by functional measurements demonstrating modification of the intended metabolite, barrier, or immune pathway (Gilbert et al., 2025).

Registered probiotic trials provide early examples of clinical translation in stroke populations. The PRISE study (ClinicalTrials.gov, identifier NCT04954846) is a randomized, double-blind, placebo-controlled trial evaluating the multi-strain probiotic OMNi-BiOTiC SR-9, administered twice daily for 3 months to patients enrolled within 7 days after acute ischemic stroke. Its primary endpoints focus on gut microbial diversity, with additional metagenomic, metabolomic, gastrointestinal, cognitive, neurological, and functional assessments. A separate single-blind randomized trial (NCT05477732) evaluates *Bifidobacterium longum* OLP-01 supplementation at 2 × 10^10^ colony-forming units per day for 12 weeks in clinically stable patients undergoing outpatient rehabilitation more than 3 months after stroke. Its outcomes include cognitive function, exercise performance, nutritional status, inflammatory markers, and gut microbial composition. These registered studies demonstrate the feasibility of testing strain-defined probiotic interventions across acute and recovery-phase stroke settings. However, their product-specific designs and predominantly microbiome-, metabolic-, and feasibility-oriented endpoints do not establish general clinical efficacy of probiotics for stroke prevention or recovery. At the registry search date of 24 July 2026, no summary results were posted for either trial, and both records were listed as having an unknown recruitment status.

### 9.3 Microbial metabolite-targeted strategies

Metabolite-targeted interventions may provide greater biological specificity than attempts to broadly remodel microbial community composition. Candidate approaches include increasing beneficial microbial functions, reducing potentially adverse metabolic pathways, directly administering microbial metabolites or derivatives, and targeting host receptors that respond to microbial products. Nevertheless, microbial source, host metabolism, dose, treatment timing, and disease phenotype must be considered.

SCFA-oriented interventions may involve dietary fiber, prebiotics, SCFA-producing bacteria, defined microbial consortia, or direct administration of acetate, propionate, butyrate, or related compounds. SCFAs can influence the epithelial barrier, regulatory T-cell differentiation, myeloid responses, endothelial function, and microglial biology. Reduced SCFA-producing bacteria and lower fecal SCFA concentrations have been associated with unfavorable outcomes after acute ischemic stroke (Tan et al., 2021). However, this association does not establish that direct SCFA supplementation will improve clinical outcome. Individual SCFAs may differ in tissue distribution, receptor activity, and biological effects, and fecal concentrations may not accurately represent epithelial or systemic exposure. Future studies should define the specific SCFA, dose, administration route, treatment window, and pharmacodynamic effect rather than treating SCFAs as a uniform protective category (Peh et al., 2022).

The TMA/TMAO pathway represents another potential therapeutic target. Gut microbial metabolism of choline, carnitine, and related nutrients generates trimethylamine, which is subsequently converted to TMAO by host enzymes. Experimental and clinical evidence links this pathway with thrombosis, vascular inflammation, ischemic stroke severity, and functional outcome, while higher circulating TMAO levels have also been associated with poor outcome after ICH (Zhu et al., 2021; Zhai et al., 2019, 2021). Potential strategies include modification of dietary substrate exposure, reduction of microbial TMA-producing capacity, and inhibition of specific microbial metabolic reactions. However, circulating TMAO is influenced by diet, renal clearance, host hepatic metabolism, comorbidity, and microbial function. It should therefore be treated as a context-sensitive biomarker and candidate mediator rather than as a universal cerebrovascular toxin. In particular, an intervention developed to modify thrombosis-related mechanisms in ischemic stroke cannot be assumed to have the same risk–benefit profile in ICH.

Bile acid and tryptophan-linked pathways are less mature but potentially relevant therapeutic domains. Microbial bile acid transformation may influence FXR and TGR5 signaling, systemic metabolism, endothelial activation, and immune regulation, and microbiota-related bile acid profiles have been associated with ischemic stroke prognosis (Wang et al., 2024b). Therapeutic studies should distinguish individual primary and secondary bile acids because their receptor activity and biological effects may differ. Similarly, tryptophan-linked pathways include microbial indole derivatives, host kynurenine metabolism, serotonin-related metabolism, and aryl hydrocarbon receptor signaling. Although tryptophan metabolites have been proposed as candidate markers of acute ischemic stroke severity and prognosis, integrated microbiome–metabolome studies are needed to distinguish microbial production from host inflammatory metabolism before these pathways can be targeted clinically (Pan et al., 2025; Qin et al., 2025).

### 9.4 Fecal microbiota transplantation and defined microbial consortia

Fecal microbiota transplantation represents a broad ecological intervention that transfers bacterial, viral, fungal, metabolic, and structural components rather than a single defined therapeutic agent. In experimental cerebrovascular research, microbiota transfer is particularly valuable for testing whether a donor microbial state can modify disease phenotype and therefore provides an important tool for causal inference. FMT remains primarily a mechanistic or preclinical strategy in cerebrovascular disease, with no established clinical indication or demonstrated therapeutic efficacy in stroke populations.

Clinical translation of fecal microbiota transplantation would require rigorous donor screening, standardized preparation and storage, defined administration routes, assessment of microbial engraftment, and prospective monitoring of infectious, metabolic, gastrointestinal, and immune adverse events. Additional challenges in acute cerebrovascular disease include antibiotic exposure, dysphagia, aspiration risk, impaired intestinal motility, enteral feeding, critical illness, and stroke-associated immunodepression. These factors may influence both the safety of administration and the ability of transferred organisms to establish stable functions.

Defined microbial consortia may provide a more controllable alternative because their strain composition and intended metabolic functions can be specified. They may also facilitate dose selection, manufacturing, reproducibility, and mechanistic validation. However, their biological effects will still depend on interactions among administered organisms, the resident microbiome, diet, medication exposure, and host physiology. FMT and defined microbial consortia should therefore remain investigational in cerebrovascular disease and should initially be evaluated through carefully designed mechanistic and early-phase safety studies rather than assumed to restore a universally “healthy” microbiome (Gilbert et al., 2025).

### 9.5 Phage-based and precision microbiome interventions

Phage-based strategies have been proposed as a means of selectively targeting bacterial strains or microbial functions while avoiding the broad ecological disruption caused by non-selective antibiotics. In principle, phages could be used to reduce pro-inflammatory bacteria, pathobionts, or organisms contributing to adverse metabolic pathways. Phage-mediated modification of bacterial populations may also indirectly alter microbial metabolic capacity and immune ligand exposure.

However, phage therapy in cerebrovascular disease remains conceptual or preclinical (Li et al., 2024). Major translational uncertainties include narrow bacterial host range, strain-level variation among patients, emergence of phage resistance, immune clearance, formulation and dosing, effects on bacterial gene transfer, and unintended changes in the wider microbial ecosystem. Bacterial lysis may also transiently alter exposure to microbial structural products, which may be particularly relevant in patients with intestinal barrier injury and systemic inflammation. Phage therapy should therefore be evaluated as an ecosystem-editing strategy rather than as a simple antibacterial intervention. Studies should document target-bacterium reduction, downstream metabolic and immune effects, ecological off-target changes, and disease-specific safety.

### 9.6 Disease- and time-window-specific translation

The clinical value and risk of microbiome-directed treatment are unlikely to be equivalent across CSVD, acute ischemic stroke, and ICH. Disease phenotype and intervention timing should therefore be incorporated into therapeutic development from the earliest experimental stage.

For CSVD, candidate interventions are most likely to be prevention-oriented and delivered over months or years. Studies should determine whether microbiome modulation slows progression of white matter hyperintensities, lacunes, cerebral microbleeds, enlarged perivascular spaces, cognitive impairment, or gait dysfunction. Short-term changes in microbial composition are insufficient endpoints for this chronic disease context. Long-term safety, adherence, medication exposure, diet, frailty, renal function, and cardiometabolic comorbidity will be particularly important.

Acute ischemic stroke requires separation of the premorbid, hyperacute, acute hospitalization, subacute recovery, and secondary prevention phases. In the hyperacute phase, reperfusion treatment and stabilization remain the primary clinical priorities, and complex microbial ecosystem reconstruction may have limited immediate feasibility. During hospitalization, infection, dysphagia, enteral feeding, aspiration risk, antibiotic exposure, gut dysmotility, and immunodepression may modify both intervention safety and microbiome measurements (Westendorp et al., 2022). Dietary, prebiotic, postbiotic, or metabolite-oriented strategies may be more feasible during recovery or secondary prevention, but their effects should be evaluated against neurological recovery, infection, functional outcome, and patient-centered measures rather than microbial composition alone.

ICH requires a hemorrhage-specific therapeutic framework. Candidate interventions should be evaluated against hematoma expansion, perihematomal edema, blood degradation product-related inflammation, white matter injury, hematoma resolution, infection, mortality, and functional recovery. Immune or metabolic modulation may have different effects during early injury and later hematoma clearance. An intervention that reduces inflammatory signaling could theoretically limit secondary injury but might also alter immune processes involved in hematoma removal or tissue repair. Similarly, pathways related to platelet activity, vascular inflammation, or thrombosis may have different implications in hemorrhagic and ischemic disease. Consequently, microbiome-directed interventions that appear beneficial in ischemic stroke should not be assumed to be safe or effective in ICH without hemorrhage-specific testing (Yu et al., 2021; Wang et al., 2024a; Jiang et al., 2024).

### 9.7 Clinical trial design, target engagement, and therapeutic endpoints

Clinical translation should follow a staged pathway. Preclinical studies should first define the microbial or metabolic target, treatment window, dose–response relationship, reversibility, and disease-specific safety profile. Early clinical studies should prioritize feasibility, tolerability, biological target engagement, and adverse-event monitoring before testing efficacy. Later-phase studies should evaluate clinically meaningful and phenotype-specific outcomes.

Therapeutic target engagement should be assessed at multiple levels. Microbial endpoints may include strain abundance, functional genes, metabolic pathways, and community resilience. Host-facing biochemical endpoints may include SCFAs, TMAO, individual bile acids, tryptophan-linked metabolites, LPS-related markers, and intestinal barrier readouts. Immune and neurovascular endpoints may include immune-cell phenotypes, cytokines, endothelial markers, BBB permeability, and imaging measures. These intermediate outcomes should then be connected to neurological, functional, cognitive, infectious, imaging, survival, or quality-of-life outcomes relevant to the specific cerebrovascular phenotype.

Baseline microbiome function may also modify treatment response. Future trials may therefore require stratification according to microbial functional deficits, metabolite profiles, antibiotic exposure, diet, renal function, nutritional status, and inflammatory phenotype. Nevertheless, biomarker-enriched designs should be externally validated and should not define patients solely by individual bacterial taxa. Clinical efficacy must ultimately be demonstrated through disease-relevant outcomes rather than inferred from increased alpha diversity, changes in selected genera, or apparent normalization of a dysbiosis index. Until such evidence is available, microbiome-directed interventions should remain investigational and should not be recommended for routine cerebrovascular treatment (Knight et al., 2018; Mirzayi et al., 2021; Gilbert et al., 2025).

## 10 Future research priorities

Future studies should move from taxonomic association toward functional, longitudinal, and mechanistic microbiome research. In CSVD, the priority is repeated MRI combined with stool metagenomics, plasma metabolomics, inflammatory profiling, and careful control of vascular and lifestyle confounders (Wardlaw et al., 2013; Duering et al., 2023; Knight et al., 2018). In acute ischemic stroke, dense early sampling over hours to days is needed to define the temporal sequence among brain injury, gut dysbiosis, barrier dysfunction, microbial translocation, immune activation, and neurological outcome (Xu et al., 2021; Błaż et al., 2024; Kumar et al., 2024). In ICH, larger longitudinal cohorts and hemorrhage-specific experimental models are required (Yu et al., 2021; Wang et al., 2024a; Jiang et al., 2024). Population-specific validation is also needed because genetic-instrument analyses suggest that microbiome–stroke associations may differ between East Asian and European populations (Cheng et al., 2024).

Across all disease contexts, multi-omics integration should be paired with causal testing. Microbiome signatures should be validated externally, linked to microbial functional pathways, connected to host immune and metabolite readouts, and tested in perturbation models. Multi-kingdom profiling, including virome and mycobiome analysis, should be incorporated into hypothesis-driven studies rather than treated as descriptive add-ons (Knight et al., 2018; Mirzayi et al., 2021; Gilbert et al., 2025).

Future therapeutic studies should follow a staged translational pathway. Preclinical experiments should define the microbial or metabolic target, treatment window, dose–response relationship, reversibility, and disease-specific safety profile. Early clinical studies should prioritize feasibility, safety, and biological target engagement before attempting to establish efficacy. Subsequent trials should connect microbial and metabolite changes with clinically meaningful neurological, imaging, cognitive, infectious, and survival outcomes. Because treatment response may depend on baseline microbial function, diet, renal function, medication exposure, nutritional status, and inflammatory phenotype, biomarker-enriched or stratified trial designs may be useful. However, proposed treatment-response biomarkers should be externally validated and should not rely exclusively on individual bacterial taxa or diversity indices.

The present review also has limitations. Because this article is a structured narrative review rather than a systematic review, study selection and interpretation may be influenced by the heterogeneity of available cerebrovascular microbiome literature. Differences in disease definitions, sampling windows, sequencing platforms, metabolomic assays, clinical confounder control, and experimental models limit direct comparison across studies. Accordingly, the evidence grading used here should be interpreted as a qualitative framework for organizing current knowledge rather than as a formal meta-analytic assessment.

## 11 Conclusion

Gut microbiome research offers a biologically plausible framework for understanding cerebrovascular vulnerability, inflammatory amplification, and recovery, but the evidence is uneven across disease contexts. Acute ischemic stroke currently provides the strongest mechanistic and clinical support for microbiome–immune–metabolic involvement. CSVD remains a biologically plausible but underdeveloped domain, while ICH is emerging and should be interpreted separately from ischemic stroke. Virome and mycobiome research remains exploratory.

A microbiology-centered interpretation should emphasize microbial ecosystem dynamics, microbial metabolites, epithelial barrier function, microbial translocation, immune programming, and neurovascular signaling. Future progress will depend on longitudinal human cohorts, multi-omics integration, disease-specific experimental models, and careful distinction between association, biomarker value, and causal mediation. Taken together, the field should move from dysbiosis-centered association toward functionally resolved, time-sensitive, and experimentally validated host–microbe mechanisms.

Therapeutic translation should proceed cautiously and should not assume that broad microbiome normalization is either achievable or beneficial. Candidate interventions must be matched to specific microbial functions, host-response pathways, cerebrovascular phenotypes, and treatment windows. At present, microbiome-directed therapies remain investigational, and routine clinical use will require disease-specific safety data, evidence of biological target engagement, standardized intervention reporting, and benefit on clinically meaningful outcomes.
