# Supplementary material for: Gut microbiome–immune–metabolic mechanisms in cerebrovascular disease: evidence-graded insights from cerebral small vessel disease, ischemic stroke, and intracerebral hemorrhage
Source: Front Microbiol. 2026 Aug 18;17:1927904. doi: 10.3389/fmicb.2026.1927904 (PMC13529723; doi:10.3389/fmicb.2026.1927904)
Supplement: Supplementary file 1 [file Table_1.docx]

Supplementary Material

**Supplementary Table 1. Key primary and methodologically relevant studies informing the review**

Note. This supplementary table is intended to increase the analytical transparency of the narrative synthesis by distinguishing direct human evidence, experimental perturbation evidence, adjacent mechanistic biology, and methodological or review-level support. Citations correspond to the references listed in the main manuscript.

| **Study** | **Domain** | **Design / model** | **Microbiome or host-response focus** | **Principal finding** | **Contribution to review framework** | **Evidence category** |
| --- | --- | --- | --- | --- | --- | --- |
| Braniste et al. (2014) | Adjacent gut-brain barrier biology | Germ-free and colonized mouse models | Microbiota status and colonization; blood-brain barrier permeability assays | Gut microbiota influenced blood-brain barrier permeability and tight-junction-related barrier regulation in mice. | Supports biological plausibility for microbiota-barrier-neurovascular links; indirect for CSVD/stroke. | Adjacent animal mechanism |
| Erny et al. (2015) | Adjacent neuroimmune biology | Germ-free and antibiotic-treated mouse models | Microbiota depletion/absence; microglial profiling and functional assays | Host microbiota regulated microglial maturation and function in the CNS. | Supports gut microbiota influence on CNS immune tone; indirect support for neuroinflammatory mechanisms. | Adjacent animal mechanism |
| Fransen et al. (2017) | Aging-related microbial ecology | Aged microbiota transfer to germ-free mice | Fecal microbiota transfer from aged donors | Aged gut microbiota contributed to systemic inflammaging-like features after transfer. | Supports the aging-microbiome-inflammatory background used in the review; not CSVD-specific. | Animal perturbation |
| Saji et al. (2021) | CSVD | Human cross-sectional study | Gut microbiome profiling and imaging-defined CSVD burden | Gut microbiome features were associated with cerebral small vessel disease burden in a cross-sectional analysis. | Direct human CSVD microbiome evidence; supports association but not temporality or causality. | Human cross-sectional |
| Fongang et al. (2023) | CSVD | Human cohort analysis within the Framingham Heart Study | Gut microbiome data and CSVD burden/neuroimaging markers | CSVD burden was associated with decreased abundance of Barnesiella intestinihominis. | Direct population-based human evidence linking gut microbial taxa to CSVD burden. | Human cross-sectional/cohort |
| Shi et al. (2023) | CSVD | Human CSVD case-control/cross-sectional study | Gut microbiota profiling and clinical/imaging evaluation | Reported altered gut microbiota in patients with CSVD and proposed clinical biomarker potential. | Adds direct human CSVD microbiome evidence; limited by observational design. | Human observational |
| Huang et al. (2024) | CSVD | Bidirectional Mendelian randomization | Genetic instruments for gut microbiota and CSVD-related neuroimaging subtypes | Suggested potential causal links between selected microbial taxa and CSVD-related imaging phenotypes. | Provides genetic-instrument evidence, but biological validation is required. | Human genetic causal inference |
| Li et al. (2019) | Adjacent CSVD metabolomics | Older-adult human cohort with brain MRI | Plasma metabolomics and MRI markers of neurodegeneration/vascular injury | Plasma metabolites were associated with brain MRI measures in older adults. | Supports metabolomic relevance to MRI-defined brain aging; not gut microbiome-specific. | Adjacent human metabolomics |
| Sliz et al. (2022) | Adjacent CSVD metabolomics | Human population metabolomics analysis | Circulating metabolome and white matter hyperintensities | Circulating metabolites were associated with white matter hyperintensity burden. | Supports metabolic links to WMH; indirect for microbiome causality. | Adjacent human metabolomics |
| Wang et al. (2025) | Adjacent CSVD metabolomics | Human plasma metabolomics and MRI analysis | Plasma metabolites, non-communicable diseases, and WMH burden | Plasma metabolites linked non-communicable disease profiles to increased WMH. | Supports metabolic vulnerability in CSVD-related MRI injury; not microbiome-specific. | Adjacent human metabolomics |
| Benakis et al. (2016) | Acute ischemic stroke | Experimental ischemic stroke model | Commensal microbiota manipulation and intestinal immune-cell profiling | Commensal microbiota influenced ischemic stroke outcome by regulating intestinal gamma-delta T cells. | Key causal experimental evidence for microbiota-immune-stroke coupling. | Animal causal mechanism |
| Singh et al. (2016) | Acute ischemic stroke | Experimental stroke model | Stroke-induced dysbiosis and neuroinflammatory response analysis | Microbiota dysbiosis controlled neuroinflammatory responses after stroke. | Supports reciprocal brain-gut-microbiome signaling after acute stroke. | Animal causal mechanism |
| Xu et al. (2021) | Acute ischemic stroke | Experimental stroke study | Stroke-induced gut dysbiosis, microbial community changes, and infarct outcome | Rapid gut dysbiosis induced by stroke exacerbated brain infarction in turn. | Supports time-sensitive bidirectional brain-gut-microbiome injury amplification. | Animal causal mechanism |
| Yamashiro et al. (2017) | Acute ischemic stroke | Human ischemic stroke study | Gut microbiota profiling with metabolic and inflammatory measures | Gut dysbiosis was associated with host metabolism and systemic inflammation in patients with ischemic stroke. | Early human evidence linking AIS dysbiosis to host-response phenotypes. | Human observational |
| Xia et al. (2019) | Acute ischemic stroke | Human stroke microbiome study | Gut microbiome-derived stroke dysbiosis index | Stroke Dysbiosis Index was associated with brain injury and prognosis of stroke. | Supports microbial signature-outcome association in AIS. | Human observational/prognostic |
| Tan et al. (2021) | Acute ischemic stroke | Human AIS cohort | Gut microbiota and fecal short-chain fatty acids | Dysbiosis and reduced short-chain fatty acids were associated with poor functional outcomes. | Key human evidence linking microbial metabolites to AIS prognosis. | Human observational/prognostic |
| Zhu et al. (2021) | Acute ischemic stroke | Experimental and translational study | Gut microbial choline/TMAO pathway and stroke severity | Gut microbes impacted stroke severity through the trimethylamine N-oxide pathway. | Mechanistic support for TMAO as a candidate microbial mediator in stroke. | Animal + translational mechanism |
| Zhai et al. (2019) | Acute ischemic stroke | Human AIS clinical study | Plasma trimethylamine N-oxide levels and outcome | Higher plasma TMAO levels had prognostic value in acute ischemic stroke. | Human clinical support for TMAO-outcome association. | Human observational/prognostic |
| Błaż et al. (2024) | Acute ischemic stroke | Human AIS clinical study | Circulating lipopolysaccharide and symptom-onset timing/outcomes | Elevated LPS was largely driven by time since symptom onset and related to clinical outcomes. | Supports microbial translocation/barrier-sensitive inflammation in AIS. | Human observational/prognostic |
| Kumar et al. (2024) | Acute ischemic stroke | Experimental stroke/gut physiology study | Enteric neuron function and gut transit after stroke | Stroke altered enteric neuron function, impaired smooth muscle relaxation, and dysregulated gut transit. | Provides physiological route for brain injury to reshape intestinal ecology. | Animal/physiological mechanism |
| Liang et al. (2025) | Acute ischemic stroke | Human oral and gut microbiome prognostic study | Oral/gut microbiota profiles and functional outcome prediction | Oral and gut microbiota features contributed to prediction of functional outcome after AIS. | Extends human prognostic evidence using oral-gut microbial signatures. | Human observational/prognostic |
| Qu et al. (2024) | Acute ischemic stroke | Mendelian randomization study | Genetic instruments for gut microbiota and functional outcome after ischemic stroke | Suggested potential links between gut microbiota and functional outcome after ischemic stroke. | Adds genetic-instrument evidence but requires biological validation. | Human genetic causal inference |
| Wang et al. (2024b) | Acute ischemic stroke | Human metabolite/prognostic study | Gut microbiota-related bile acids and clinical prognosis | Bile acid profiles were associated with clinical prognosis in ischemic stroke patients. | Supports bile acids as emerging microbial metabolite pathway in AIS. | Human observational/prognostic |
| Pan et al. (2025) | Acute ischemic stroke | Human metabolomics/prognostic study | Tryptophan metabolites and AIS severity/prognosis | Tryptophan metabolites were proposed as biomarkers for AIS severity and prognosis. | Supports tryptophan-linked metabolites as emerging host-facing mediators. | Human observational/prognostic |
| Yu et al. (2021) | Intracerebral hemorrhage | Experimental ICH model | ICH-induced gut microbiota dysbiosis and neuroinflammation | Gut microbiota dysbiosis induced by ICH aggravated neuroinflammation in mice. | Key animal evidence that hemorrhagic brain injury can interact with gut dysbiosis. | Animal mechanism |
| Wang et al. (2024a) | Intracerebral hemorrhage | Human ICH clinical sample study | Gut microbiota dysbiosis and neurological recovery after ICH | Gut microbiota dysbiosis was associated with neurological function recovery after ICH. | Direct human ICH microbiome evidence; supports clinical association. | Human observational |
| Jiang et al. (2024) | Intracerebral hemorrhage | Human pilot study | Gut microbiome variation in early-stage mild-to-moderate ICH | Reported gut microbiome variation in early ICH and explored potential therapeutic targets. | Adds early human pilot evidence for ICH-specific microbiome changes. | Human pilot observational |
| Zhai et al. (2021) | Intracerebral hemorrhage | Human ICH clinical study | Plasma trimethylamine N-oxide levels and outcome | High plasma TMAO levels were associated with poor outcome in ICH patients. | Supports metabolite-focused clinical association in ICH. | Human observational/prognostic |
| Xiao et al. (2022) | Intracerebral hemorrhage | Experimental ICH mouse study | NLRP3 inflammasome targeting, gut microbiota, corticospinal tract injury | Targeting NLRP3 modulated gut microbiota and attenuated injury/deficits after ICH. | Supports hemorrhage-specific microbiota-inflammasome-neuroinjury pathway. | Animal mechanism/intervention |
| Li et al. (2023) | Intracerebral hemorrhage | Experimental ICH mouse study | Oxymatrine intervention, gut microbiota, white matter injury | Oxymatrine ameliorated white matter injury by modulating gut microbiota after ICH in mice. | Links gut microbiota to white matter injury in hemorrhagic brain injury models. | Animal mechanism/intervention |
| Zheng et al. (2025) | Intracerebral hemorrhage | Experimental ICH study | Gut microbiota depletion, p-coumaric acid, Treg differentiation, hematoma resolution | Gut microbiota depletion accelerated hematoma resolution and recovery via p-coumaric acid-promoted Treg differentiation. | Supports microbiota-metabolite-immune pathway in hematoma resolution. | Animal mechanism/intervention |
| Wang et al. (2021) | Virome/phage | Human stroke virome/microbiome study | Gut virome and microbiome characteristics in patients with stroke | Reported altered gut viral/microbial features in stroke patients. | Early direct human virome evidence; exploratory. | Human exploratory |
| Chelluboina et al. (2022) | Virome/phage | Experimental focal cerebral ischemia model | Gut virome profiling after focal cerebral ischemia | Focal cerebral ischemia was associated with gut virome dysbiosis in mice. | Supports stroke-responsive virome changes; exploratory mechanistic domain. | Animal exploratory |
| Underhill and Iliev (2014) | Mycobiome/adjacent immune biology | Immunology review of commensal fungi | Synthesis of fungal-host immune interactions | Commensal fungi can interact with host immune pathways and pattern-recognition systems. | Provides adjacent rationale for mycobiome discussion; not cerebrovascular-specific. | Adjacent review-level support |
| Gacesa et al. (2022) | Microbiome confounding/methodology | Large population gut microbiome study | Environmental, dietary, medication, and health-related determinants of gut microbiome composition | Environmental and host factors strongly shaped gut microbiome variation. | Supports need to control diet, medication, health status, and other confounders. | Population microbiome methodology |
| Knight et al. (2018) | Microbiome methods | Best-practices methodological article | Microbiome analysis workflow considerations | Outlined best practices for microbiome study design, analysis, and interpretation. | Supports methodological recommendations for sequencing, analysis, and reproducibility. | Methodological guidance |
| Mirzayi et al. (2021) | Microbiome reporting | STORMS reporting guideline | Human microbiome reporting checklist | Provided reporting standards for human microbiome research. | Supports transparent reporting of sample collection, storage, sequencing, analysis, covariates, and data availability. | Reporting guideline |
| Gilbert et al. (2025) | Microbiome translation | Translational microbiome review | Clinical translation of microbiome research | Highlighted opportunities and limitations in translating microbiome findings into clinical use. | Supports cautious framing of diagnostics, interventions, and therapeutic readiness. | Review-level translational guidance |

Abbreviations: AIS, acute ischemic stroke; BBB, blood-brain barrier; CNS, central nervous system; CSVD, cerebral small vessel disease; ICH, intracerebral hemorrhage; LPS, lipopolysaccharide; MRI, magnetic resonance imaging; SCFA, short-chain fatty acid; TMAO, trimethylamine N-oxide; WMH, white matter hyperintensity.
